# The Polish version of the Ethical Climate Questionnaire

**DOI:** 10.1038/s41598-025-93990-7

**Published:** 2025-03-14

**Authors:** Marcin Wnuk, Agnieszka Czerw, Marta Żywiołek-Szeja, Agata Chudzicka-Czupała

**Affiliations:** 1 Faculty of Psychology and Cognitive Sciences, Adam Mickiewicz University in Poznan, Wieniawskiego 1, 61-712 Poznan, Poland; 2 Faculty of Psychology and Law in Poznań, Interdisciplinary Center for Social Activity & Well-Being Research FEEL & ACT WELL, SWPS University, Poznan, Poland; 3 Faculty of Psychology in Katowice, Interdisciplinary Center for Social Activity & Well-Being Research FEEL & ACT WELL, SWPS University, Katowice, Poland

**Keywords:** The Ethical Climate Questionnaire, Measure, Confirmatory factor analysis, Convergent validity, Discriminant validity

## Abstract

Ethical organizational climate is a pivotal topic in business ethics, influencing employees’ well-being and attitude toward the organization. In Poland, there is a lack of measures dedicated to verifying organizational climate. Our research aimed to examine the psychometrical properties of the Ethical Climate Questionnaire (ECQ) in Polish business conditions. Studies conducted on two samples confirmed the original structure of the Polish version of the ECQ and its better psychometric properties compared to the original version. Confirmatory factor analysis (CFA) confirmed good construct validity and internal consistency of the ECQ, with Cronbach’s alpha coefficients in the range of 0.74–0.81, depending on the type of ethical climate. Bagozzi and Heatherton’s criteria of the magnitude of factor loadings, average variance extracted, and composite reliability showed good convergent validity of the tool. The value of the heterotrait–monotrait ratio of correlations indicated good discriminant validity of the ECQ. Also, the criterion validity of this measure was confirmed. In conclusion, the Polish version of the ECQ is a measure that can be used to study the ethical climate in Polish organizations.

## Introduction

The issue of ethical business, including the ethical activities of organizations as institutions, as well as employees’ ethical behavior, has long been present in work and organizational psychology^1^. This perspective addresses specific actions or behaviors. However, the perspective of employees’ subjective assessment of the ethicality of their organization seems to be underappreciated and much less explored, especially in the Polish context. Employees’ subjective feelings about the way the organization functions are usually operationalized as organizational climate. A search in the Ebsco database regarding research on ethical organizational climate in the Polish context returned only seven articles on this topic, published in 2013–2023. It is worth noting that the authors usually resorted to translated versions of the questionnaires^2,3^. To our best knowledge, there is no psychometrically verified Polish version of the method for diagnosing ethical climate. At a time of increasing interest in creating positive organizations and looking for effective ways to take care of employee well-being, it is surprising that there is so little interest in the ethical climate in Polish science and organizational practice. One of the reasons for this situation may be precisely the lack of a well-validated questionnaire. Consequently, the study presenting the psychometric properties of the Polish version of the Ethical Climate Questionnaire^4^ is a significant contribution to conducting reliable research in a Polish and in an intercultural context. The tool has gained a prominent place in empirical research on business ethics. Meta-analyses show that the ethical climate that the tool measures is linked to important organizational outcomes^5^. Although some authors^6,7^ discuss its certain features and consider the practical applicability of the questionnaire to be limited, its advantages and the fact that it is commonly used in many studies make it worth adapting to the Polish conditions.

Organizational climate, or work climate, in its broadest definition, means employees’ shared perceptions of psychologically important characteristics of procedures and processes, both formal and informal, implemented in the organization^4,8^. Ethical climate is defined as the perception of what constitutes appropriate behavior in a work situation, and thus becomes the psychological mechanism by which ethical issues are managed in the organization^5,8^. Ethical climate theory was developed by Victor and Cullen^4^. The authors took into account Kohlberg’s theory of moral development and Schneider’s work on sociocultural theories of organization^8^. Three ethical categories are taken from Kohlberg’s theory: egoism (maximizing one’s benefits, and self-interest), benevolence (maximizing the interests of as many people as possible), and principles (adhering to universal standards and beliefs). These ethical categories are associated with three reference groups that constitute the sources of moral reasoning, i.e. what is considered ethically appropriate, namely: individual (based on the employee’s self-defined ethical beliefs); local (based on the organization’s standards and principles); and cosmopolitan (external to the individual and the organization, for instance coming from a professional association). The combination of three ethical standards with three references creates nine theoretical dimensions of the ethical work climate^4,9^. However, empirical research does not confirm the existence of these nine climate types. Both the questionnaire created by the authors of the ECQ^4^ and other analyses^8^ usually indicate five types of ethical climate: instrumental, caring, independence, law and code, and rules.

The* instrumental climate* is characterized by an emphasis on maximizing self-interest at the individual or organizational level. Employees perceive decisions in the organization as being made from the egoistic perspective of the manager pursuing their own or the organization’s interests. This also happens despite the negative consequences for individual employees or employee groups. The main operating principle is the good of the organization as a formal institution. The *caring climate* emphasizes the well-being of others. Employees feel that the organization’s decisions and actions take into account their interests, not just those of the organization as such. The belief is that taking care of employees serves the organization as a community. The main rule in the organization is kindness towards all employees. The *independence climate* focuses on valuing individual ethical beliefs. The organization ensures that each employee can make ethical decisions based on their principles. The influence of external rules is minimized. The organization leaves employees free to assume ethical responsibility for their conduct. The basic principle is trust in the employees’ moral and ethical correctness. The *law and code climate* emphasizes compliance with general legal regulations and professional standards. The organization operates on the basis of the law in force in a given country, region, etc., and on the existing standards of functioning in a given profession, if any. Employees see their organization as paying special attention to compliance with external legal rules. The main principle of the organization’s operation is compliance with the law. The *rules climate* is characterized by an emphasis on local rules and procedures. The organization ensures that all employees know and precisely apply the rules set forth in the internal regulations, such as statutes, codes, and various bylaws. The main principle of operation in the organization is the superiority of organizational rules over other rules of conduct. It is worth noting that the five types of ethical climate are not always treated separately. In some studies and analyses, they are ranked from the least to the most ethical^10^ considering the types of ethical criteria and levels of analysis. In this case, the order will go from a cosmopolitan climate (law and code) to a climate of individual interests (instrumental). This approach enables analyses taking into account the ethical climate as a mediator between other variables. However, most analyses are based on separate treatments of climate types.

Many studies show that these five types of climate are differentially associated with both positive and negative consequences. The research mainly concerns employee consequences understood as attitudes towards work and organization, but also as specific behaviors. Newman and colleagues^11^ point to four groups: work attitudes (e.g., job satisfaction, organizational commitment), ethical outcomes (e.g., ethical intentions, ethical behavior), psychological states (e.g. moral distress, mindfulness), and performance outcomes (e.g. job performance, teamwork). A review of the research on ethical climate correlates makes it possible to indicate that positive outcomes are especially related to caring climate: job satisfaction^5,8,11–13^, organizational commitment^5,8,11,12,14,15^, and organizational citizenship behavior^10–12^. Another clear conclusion from the research review is the positive correlation of instrumental climate (sometimes referred to as egoistic) with negative outcomes such as workplace deviant behavior^9^, bullying behavior^16^, turnover intention^5,11,17^, job burnout, and in particular emotional exhaustion^17^. Most often, research shows that a caring climate has the opposite effects to an instrumental climate. The conclusions from correlation analyses of other climate types are slightly less obvious. Typically, however, the two types of climate related to compliance with general legal or organizational provisions and principles (respectively: law and code, and rules) have predominantly positive consequences^5,11,18^.

The study aims to analyze the psychometric properties of the Ethical Climate Questionnaire^4^ in the Polish context. Based on the above-mentioned conclusions from other studies, we assume that the instrumental climate will negatively correlate with the well-being indicators used in this study, and positively with stress. However, the caring climate will show opposite relationships. Another important goal is to fill the gap in investigating this area in the realities of Polish organizations, which will be possible thanks to the adaptation of the questionnaire.

## Method and results

Our study was a methodological cross-sectional survey. It was conducted on two samples of full-time employees working in different companies in Poland. The different stages of the analysis were aimed at translating the tool and determining its internal validity, convergent validity, discriminant validity, and reliability in the Polish employee population.

We used the back-translation method to prepare the Polish version of the measure^19^. In the first stage, three specialists, translators who were also psychologists and academic teachers, made their translations from English to Polish. Next, three other translators made their back-translations. After comparing the texts obtained, the final version was defined.

The present study was approved by the Ethics Committee of SWPS University, Katowice, Poland (Ethics code: WKEB89/11/2023). All ethical principles, including obtaining necessary permission from the main designers of the measure, obtaining written informed consent from all participants, ensuring the confidentiality of their information, and freedom to withdraw from the study, were observed at every step.

### Study 1: internal validity, reliability, and discriminant validity of the ECQ

#### Participants

The research encompassed 434 full-time employees with employment contracts in different companies in Poland. Every participant expressed written consent to participate in the study. The sample was balanced on the grounds of sex and education. It included 252 women (58.6%) and 178 men (41.4%). The mean age of the participants was M = 29.58 years (SD = 10.79), with mean seniority of M = 8.66 years (SD = 9.5). Four (0.9%) of them had elementary education, and seven participants (1.6%) had vocational education. A total of 180 participants (41.9%) had completed secondary, and 239 (55.6%) higher education. In terms of the position held, 260 participants (43.5%) were ordinary workers, 319 employees (34.2%) were independent specialists, 57 participants (7%) were low-level managers, 86 respondents (11.9%) were mid-level managers, and 32 respondents (3.5%) were senior-level managers.

#### Internal validity and reliability of the ECQ

Confirmatory factor analysis (CFA) was used as an appropriate method to verify whether the original five-factor structure of the ECQ^4^ would be reflected in data from a Polish employee population.

Results showed that the five-factor model of the ECQ is poorly fitted: CMIN/DF 2.84 (*p* = 0.000), RMSEA [0.065. 90% (0.060; 0.071)], SRMR = 0.0784, CFI = 0.88, TLI = 0.85, NFI = 0.88, GFI = 0.88. Additionally, six items of the ECQ, three as caring ethical organization climate indicators and three as instrumental ethical organization climate indicators loaded these factors below the threshold level of 0.4 (see Table 1) established as a minimum^20,21^. After deleting these items from further analysis, CFA revealed that the five-factor solution was well fitted to the data (see Table 2), and every item loaded higher than 0.4^20,21^, with Cronbach’s alpha coefficient as a reliability indicator being above the acceptable level of 0.7^22^ for every ECQ dimension (see Table 1). It indicated that the five-factor original version of the ECQ, consisting of 20 items, has satisfactory internal validity and reliability.

**Table 1:** CFA and Cronbach’s alpha results of the ECQ (n = 434).

Items English version	Items Polish version	Factor	Estimate original version	Estimate revised version	Cronbach’s alpha
What is best for everyone in the company is the major consideration here	To, co jest najlepsze dla wszystkich w firmie, jest tutaj najważniejszą kwestią	Caring	.804	.805	.79
The most important concern is the good of all the people in the company as a whole	Przedmiotem najwyższej troski jest dobro wszystkich ludzi w firmie jako całości		.836	.846	
Our major concern is always what is best for the other person	Naszą główną troską jest zawsze to, co jest najlepsze dla drugiej osoby		.736	.735	
In this company, people look out for each other’s good	W tej firmie ludzie czynią sobie wzajemnie dobro		.523	.518	
In this company, it is expected that you will always do what is right for the customers and public	W tej firmie zawsze oczekuje się zrobienia tego, co jest właściwe dla klientów i opinii publicznej		− .004		
The most efficient way is always the right way in this company	Najbardziej efektywny sposób jest zawsze właściwy w tej firmie		.164		
In this company, each person is expected above all to work efficiently	W tej firmie oczekuje się przede wszystkim efektywnej pracy od każdej osoby		− .014		
People are expected to comply with the law and professional standards over and above other considerations	Od ludzi oczekuje się tu przede wszystkim przestrzegania prawa i standardów zawodowych	Law and code	.613	.623	.77
In this company, the law or ethical code of their profession is the major consideration	W tej firmie głównym przedmiotem rozważań jest prawo lub kodeks etyczny ich zawodu		.568	.577	
In this company, people are expected to strictly follow legal or professional standards	W tej firmie oczekuje się od ludzi ścisłego przestrzegania norm prawnych i standardów zawodowych		.798	.799	
In this company, the first consideration is whether a decision violates any law	W tej firmie najważniejszą kwestią do rozważenia jest to, czy decyzja narusza jakiekolwiek prawo		.652	.645	
It is very important to follow the company’s rules and procedures here	Przestrzeganie zasad i procedur obowiązujących w firmie jest bardzo ważne	Rules	.682	.830	.81
Everyone is expected to stick by company rules and procedures	Od każdego oczekuje się przestrzegania zasad i procedur firmy		.793	.792	
Successful people in this company go by the book	Ludzie sukcesu w tej firmie stosują się do obowiązujących zasad		.637	.512	
People in this company strictly obey the company policies	Osoby w tej firmie ściśle przestrzegają obowiązujących zasad		.872	.836	
In this company, people protect their own interests above all else	W tej firmie ludzie nade wszystko chronią swoje własne interesy	Instrumental	.730	.714	.74
In this company, people are mostly out for themselves	W tej firmie ludzie działają głównie dla siebie		.810	.848	
There is no room for one’s own personal morals or ethics in this company	W tej firmie nie ma miejsca na własną moralność czy etykę		.549	530	
People are expected to do anything to further the company’s interests regardless of the consequences	Oczekuje się, że ludzie zrobią tu wszystko, aby wspierać interesy firmy, bez względu na konsekwencje		.443	.409	
People here are concerned with the company’s interests—to the exclusion of all else	Ludzie troszczą się o interesy firmy—wykluczając tym samym wszystkie inne sprawy		.207		
Work is considered substandard only when it hurts the company’s interests	Praca jest uważana za niespełniającą norm tylko wtedy, gdy szkodzi interesom firmy		.364		
The major responsibility of people in this company is to control costs	Głównym obowiązkiem ludzi w tej firmie jest kontrola kosztów		.396		
In this company, people are expected to follow their own personal and moral beliefs	W tej firmie oczekuje się, że ludzie będą postępować zgodnie z własnymi przekonaniami osobistymi i moralnymi	Independence	.744	.740	.77
Each person in this company decides for themselves what is right and wrong	Każda osoba w tej firmie sama decyduje, co jest dobre, a co złe		.471	.470	
The most important concern in this company is each person’s own sense of right and wrong	Przedmiotem najwyższej troski w tej firmie jest własne poczucie dobra i zła każdej osoby		.774	.772	
In this company, people are guided by their own personal ethics	W tej firmie ludzie kierują się swoją osobistą etyką		.597	.578

**Table 2:** Results of fit indices indicators of different models of ECQ (n = 434).

Number of model	Factors	CMIN/DF	RMSEA	SRMR	CFI	TLI	NFI	GFI
Five factor model	CR, LAC, R, INST, I	1.2 (*p* = 0.064)	[0.022. 90% (0.000; 0.033)]	0.043	0.99	0.96	0.99	0.97
Four factor model	CR, LAC + R, INST, I	1.93 (*p* = 0.000)	[0.046. 90% (0.038; 0.055)]	0.053	0.96	0.93	0.95	0.95
Three factor model	CR + LAC + R. INST, I	6.34 (*p* = 0.000)	[0.111. 90% (0.104; 0.111)]	0.110	0.79	0.69	0.76	0.81
Two factor model	CR + LAC + R + INST, I	7.74 (*p* = 0.000)	[0.125. 90% (0.118; 0.132)]	0.113	0.74	0.62	0.71	0.78
One factor model	CR + LAC + R + INST + I	9.43 (*p* = 0.000)	[0.139. 90% (0.132; 0.147)]	0.123	0.67	0.52	0.65	0.74

Caring = CR; Law and code = LAC; Rules = R; Instrumental = INST; Independence = I.

The five types of organizational climate explained 63.34% of the variance of this construct, respectively: law and code ethical organizational climate—28.17%, caring ethical organizational climate—14.87%, independence ethical organizational climate—8.83%, rules ethical organizational climate—6.36% and instrumental ethical organizational climate—5.09%.

Additionally, the fitness of alternative models with one, two, three, and four factors was tested. Only the model with four factors had a good adjustment to the data, but in comparison with the five-dimensional model, its fitness was statistically significantly worse (see Table 2).

#### Discriminant validity

Discriminant validity was examined by using the heterotrait-monotrait ratio of correlations (HTMT) method with a threshold value of 0.9^23^ and correlations between factors encompassing the ethical organizational climate construct, whose values should be less than 0.8^24^.

As presented in Table 3, the HTMT ratio did not exceed 0.9 for any of the pairs of ethical organizational climate types. Also, correlations between them were lower than 0.8. Caring climate correlated positively with law and code climate (r = 0.25; *p* < 0.01), rules climate (r = 0.36; *p* < 0.01), and independence climate (r = 0.53; *p* < 0.01), and negatively with instrumental climate (r =  − 0.48; *p* < 0.01). Law and code climate was positively related to rules climate (r = 0.72; *p* < 0.01) and independence climate (r = 0.2; *p* < 0.01), and negatively to instrumental climate (r =  − 0.16; *p* < 0.01). Rules climate correlated positively with independence climate (r = 0.11; *p* < 0.05) and negatively with instrumental climate (r =  − 0.32; *p* < 0.01), which in turn was negatively related to independence climate (r =  − 0.24; *p* < 0.01).

**Table 3:** Results of heterotrait–monotrait ratio of correlations (n = 434).

Types of organizational ethical climate	Monotrait	Heterotrait		HTMT ratio	
		Pair of variables		Pair of variables	
CR	.531	CR–LAC	.131	CR–LAC	.264
LAC	.461	CR–R	.228	CR–R	.850
R	.506	CR–INST	.248	CR–INST	.528
INST	.417	CR–I	.255	CR–I	.529
I	.437	LAC–R	.359	LAC–R	.743
		LAC–INST	.045	LAC–INST	.103
		LAC–I	.066	LAC–I	.147
		R–INST	.159	R–INST	.347
		R–I	.063	R–I	.134
		INST–I	.117	INST–I	.275

Caring = CR; Law and code = LAC; Rules = R; Instrumental = INST; Independence = I.

### Study 2: internal and convergent validity of the ECQ

#### Participants

The study participants were 1,071 employees from different organizations located in Poland. All of them gave written consent to participate in the study. Most of them were women (860; 80.3%), 200 were men (18.7%), and 11 declared another gender (1%). The mean age of the participants was M = 28.64 years (SD = 8.74), with mean seniority of M = 7.88 years (SD = 7.04). Two (0.2%) of the participants had elementary education, four (0.4%) had vocational education, 539 (50.3%) had secondary education, and 526 (49.1%) had higher education. A total of 432 (40.3%) were ordinary workers, 412 (38.5%) were independent specialists, 50 (4.7%) were low-level managers, 106 (9.9%) were mid-level managers, and 71 (6.6%) were senior-level managers. A total of 278 participants (26%) were employed in micro-organizations, 254 (23.7%) in small companies, 152 (14.2) in medium-sized companies, and 387 (36.1%) in large companies.

#### Internal validity

CFA was conducted on a 20-item version of the ECQ for five variants of the model, respectively for one-factor, two-factor, three-factor, four-factor, and five-factor solutions. The results presented in Table 4 show that only the five-factor model following the original structure of this measure has a good fit to the data. The five-factor solution explained 70.98% of the variance of ethical organizational climate, respectively: law and code ethical climate—31.75%, independence climate—17.23%, caring climate—10.88%, rules climate—6.56%, and instrumental climate—4.5%.

**Table 4:** Results of fit indices indicators of different models of ECQ (n = 1071).

Number of model	Factors	CMIN/DF	RMSEA	SRMR	CFI	TLI	NFI	GFI
Five factor model	CR, LAC, R, INST, I	3.3 (*p* = 0.000)	[0.047. 90% (0.040; 0.053)]	0.035	0.99	0.97	0.98	0.98
Four factor model	CR, LAC + R, INST, I	10.59 (*p* = 0.000)	[0.095. 90% (0.089; 0.0101)]	0.057	0.95	0.87	0.94	0.93
Three factor model	CR + LAC + R. INST, I	18.93 (*p* = 0.000)	[0.130. 90% (0.124; 0.136)]	0.116	0.9	0.76	0.9	0.81
Two factor model	CR + LAC + R + INST, I	27.97 (*p* = 0.000)	[0.160. 90% (0.154; 0.165)]	0.142	0.85	0.64	0.85	0.76
One factor model	CR + LAC + R + INST + I	38.83 (*p* = 0.000)	[0.189. 90% (0.183; 0.195)]	0.156	0.79	0.49	0.79	0.76

Caring = CR; Law and code = LAC; Rules = R; Instrumental = INST; Independence = I.

Just like in Study 1, every item loaded the factor higher than the established standard of 0.4^20,21^, and only one item loaded the factor lower than 0.6 (see Table 5).

**Table 5:** Results of methods used to verify convergent and divergent validities and composite reliability (n = 1071).

Indicator variables	Latent variables	Standardized loadings	Square of standardized loadings	Sum of square of standardized loadings	Average variances extracted (AVE)	Square of (AVE)	Composite reliability
1	Caring	.795	63.20	216.93	54.23	0.74	.87
2		.736	54.17				
3		.657	43.16				
4		.751	56.40				
5	Law and code	.450	20.25	235.19	58.79	.77	.86
6		.844	71.23				
7		.881	77.61				
8		.813	66.10				
9	Rules	.618	38.19	203.07	50.92	.71	.86
10		.642	63.68				
11		.842	42.22				
12		.798	58.98				
13	Instrumental	.645	41.60	182.2	45.55	.67	.79
14		.718	51.55				
15		.677	45.83				
16		.662	43.82				
17	Independence	.691	47.74	213.82	53.45	.73	.86
18		.841	70.72				
19		.698	48.72				
20		.683	46.64				

Besides Bagozzi & Heatherton’s^25^ criterion of convergent validity regarding the magnitude of most factor loadings (threshold 0.50), which was fulfilled, two other conditions for convergent validity suggested by these authors were met, such as composite reliability (qC) (threshold 0.60–0.70) and average variance extracted (threshold 0.50), but with one exception, that of instrumental ethical organizational climate (see Table 5).

Composite reliability, also known as construct reliability, was higher than the acceptable standard^26^ for all dimensions of the Polish version of the ECQ (see Table 3). In contrast to Cronbach’s alpha coefficient, the composite reliability coefficient is free from measurement error. The differences in outcomes between these methods were not large and both of them confirmed that the Polish version of the ECQ has satisfactory reliability (see Table 5).

Although the average variance extracted for the instrumental climate was close to, but below the threshold level, the composite reliability for this dimension was higher than the acceptable standard 0.7, which means that following Fornell and Larcker^27^, it can be admitted that the condition for convergent validity was met.

#### Divergent validity

The HTMT ratio was applied to test divergent validity. As presented in Table 6, for every pair of ECQ dimensions, the HTMT value is below the established standard, which means that this measure characterizes satisfactory divergent validity. This conclusion is supported by the values of the correlation coefficients between the ECQ factors, which did not exceed 0.8.

**Table 6:** Results of heterotrait–monotrait ratio of correlations (n = 1071).

Types of organizational ethical climate	Monotrait	Heterotrait		HTMT Ratio	
		Pair of variables		Pair of variables	
CR	.534	CR–LAC	.133	CR–LAC	.269
LAC	.453	CR–R	.224	CR–R	.824
R	.509	CR–INST	.22	CR–INST	.460
INST	.428	CR–I	.269	CR–I	.553
I	.443	LAC–R	.368	LAC–R	.766
		LAC–INST	.045	LAC–INST	.102
		LAC–I	.082	LAC–I	.183
		R–INST	.135	R–INST	.289
		R–I	.090	R–I	.190
		INST–I	.103	INST–I	.238

Caring = CR; Law and code = LAC; Rules = R; Instrumental = INST; Independence = I.

#### Criterion-related validity

Four measures of criterion validity were used: perceived stress at work, job satisfaction, turnover intention, and meaning in work.

The Polish version of the Perceived Stress Scale (PSS-10) was used to diagnose the feeling of stress at work. The Polish adaptation^28^ is based on the scale invented by Cohen and colleagues^29^. It is a one-dimensional scale consisting of 10 items. Respondents answer on a five-point frequency scale: from 1—never, to 5—very often. They refer to their feelings at work from the past month. Cronbach’s α coefficient in this study was 0.75.

Job satisfaction was measured using one statement: “How generally are you satisfied with work”. Participants responded by choosing on a ten-point scale between (1) very dissatisfied to very satisfied (10).

The turnover intention was examined using a three-item measure^30^. The item contents were as follows: “*I intend to leave the organization*”, “*I intend to make a genuine effort to find another job over the next few months*”, and “*I often think about quitting*”. Participants responded on a 5-point Likert scale ranging from 1 = strongly disagree to 5 = strongly agree. This measure has good psychometric properties. In this study, the reliability of the scale, assessed by Cronbach’s α coefficient, was 0.82.

The Work and Meaning Inventory in the Polish version, i.e. WAMI-PL^31^ was used to assess the sense of meaning in work. The questionnaire was inspired by Steger’s WAMI^32^. The Polish version consists of two dimensions: Meaning of work in the self-perspective (six items) and Meaning of work in the world perspective (four items). In this study, we used only items regarding the meaning of work in the self-perspective. The reliability of the measure, assessed by Cronbach’s α coefficient, was 0.89.

Criterion validity was examined through the correlation between types of ethical organizational climate and well-being and ill-being measures. The results of correlation coefficient values are shown in Table 7.

**Table 7:** Correlation coefficient results between the ECQ and measures used for criterion validity (n = 1071).

	Caring	Law and code	Rules	Instrumental	Independence
Caring					
Law and code	0.35**				
Rules	0.38**	0.70**			
Instrumental	− 0.35**	− 0.06	− 0.15**		
Independence	0.48**	0.16**	0.14**	− 0.20**	
Stress at work	− 0.32**	− 0.11**	− 0.14**	0.38**	− 0.14**
Job satisfaction	− 0.04	0.08**	0.04	0.07*	− 0.06
Turnover intentions	− 0.48**	− 0.21**	− 0.25**	0.39**	− 0.33**
Meaning in work	0.43**	0.23**	0.22**	− 0.22**	0.32**
Age	− 0.07*	0.03	0.01	− 0.10**	− 0.04
Education	− 0.10**	− 0.06*	− 0.06*	− 0.06*	− 0.01
Overall seniority	− 0.05	− 0.02	− 0.02	0.01	0.01
Seniority in current organization	− 0.05	− 0.03	− 0.03	0.02	0.01
Level the position held	0.09**	0.05	0.07*	− 0.06*	0.10*
Company size	− 0.22**	0.07*	0.10**	0.16**	− 0.23**

*p < 0.05. **p < 0.01.

The caring, law and code, and rules ethical climates were negatively related to stress, turnover intentions, and education as well as positively related to meaning in work. The independence ethical climate was negatively related to stress, turnover intentions, and organizational size as well as negatively related to meaning in work, and the position held. The instrumental ethical climate correlated positively with stress and turnover intentions and negatively with job satisfaction, meaning in work, education, and the level of the position held. Additionally, the level of the position held was positively related to the caring and rules ethical climates, whereas organizational size correlated negatively with the caring ethical climate and negatively with the law and code and rules climates.

#### Analyses with a demographic variable: gender

Additionally, gender differences were examined. According to the obtained results, women and men differ in the level of independence ethical climate t(1,058) = 2.29, *p* < 0.05; *p* < 0.01; Men M = 10.57, SD = 3.75, Women M = 11.23; SD = 3.6) but in terms of the law and caring, law and code, rules, and instrumental climates, no difference was detected, with respectively t(1,058) = 1.69, *p* = 0.09; Men M = 11.88, SD = 3.83, Women M = 12.38; SD = 3.78), t(1,058) =  − 0.05, *p* = 0.47; Men M = 13.33, SD = 4.01, Women M = 13.31; SD = 3.76), t(1,058) = 0.22, *p* = 0.41; Men M = 14.25, SD = 3.63, Women M = 14.31; SD = 3.64), and t(1,058) =  − 1.24, *p* = 0.11; Men M = 11.43, SD = 3.47, Women M = 11.08; SD = 3.6). Women presented a higher level of independence climate than men.

## Discussion

Our study aimed to examine the psychometric properties of the ECQ^4^ in a sample of Polish employees as a potentially reliable and valid measure to use in the research of the organizational ethical climate in Polish socioeconomic and business conditions. As previous studies confirmed, the ethical climate is a pivotal topic for social good defined from the perspectives of benefits of employees and companies^5,8,11^. Recent studies in several countries have supported the assumption that ethical climate perception is a good predictor of different well-being indicators and desirable attitudes in the workplace, such as job satisfaction, organizational commitment, turnover intentions, and ethical and dysfunctional behavior^5,8,11,33–36^. However, such research is lacking in Poland. With the Polish version of the questionnaire, it will be possible to check whether the above-mentioned relationships are also confirmed in the Polish reality. We are aware that the study presented here is only a trigger for larger-scale research. For example, a study of different industries, and organizations varying in size or type of ownership, will provide further insight into whether such relationships are universal or specific to certain environments. Knowledge of this will allow for a better understanding of how to improve employee well-being and stimulate behavior useful to the organization. This can subsequently be translated into practical actions implemented in organiz ations.

As suggested by findings from studies conducted in other countries, in this study, we used certain correlates of ethical climate as validity criteria (e.g., job satisfaction, turnover intentions, stress at work), but we also added the sense of meaning in work. This is an important indicator of well-being that has not previously been used in ethical climate research. Like in earlier studies, the same tendency is revealed, namely that the instrumental climate was negatively connected with beneficial outcomes, such as meaning in work, and positively with detrimental and undesirable outcomes, such as stress and turnover intentions. Employees who assess the organizational climate as instrumental are more motivated to quit their jobs^35,36^, are more stressed, and have a problem with finding meaning in work.

In turn, in the same vein as in previous studies, the remaining ethical climate types were positively related to profitable consequences and negatively correlated with harmful ones. This means that the caring, rules, law and codes, as well as independence ethical climates, play similar positive roles in employees’ well-being and their attitude toward their companies, facilitating finding meaning in work and protecting from stress and turnover intentions^35,36^.

Only one discrepancy with recent research was found. The caring and instrumental ethical climates were both irrelevant to job satisfaction^13,33,35–37^. One of the reasons for this could be the specificity of the measure used to examine job satisfaction, which was one general question.

According to the study findings, the perception of the ethical organizational climate among Polish employees is independent of seniority but partially depends on age, gender, education, and level of position held. Older employees have a lower tendency to perceive caring and instrumental climates, but the perception of other types of ethical climates is unrelated to age. Women scored higher in the independence ethical climate, which means that they are more prone to see the organizational climate through the prism of their values, norms, and codes of conduct. On the other hand, the independence climate was the only type of ethical climate unrelated to education, but the remaining climate types were negatively correlated with education. This implies that more educated individuals perceive the ethical rules contained in organizational procedure and politics from a more individual and autonomous point of view, and follow organizational cues and suggestions less often. A higher position in the organization goes hand in hand with the positive perception of the caring, rules, and independence climates, and a negative perception of the instrumental climate, but is irrelevant to the law and code climate.

One way to explain these results may be the role played by managers as organizational representatives responsible for developing and distributing ethical rules, principles, and standards. What is essential is that the bigger the company is, the stronger the perception of the law and code, rules, and instrumental climates, and the weaker the caring and independence climates are perceived by employees. This shows two interesting phenomena. The first one is that individual moral standards, values, and rules have a less significant position and less meaning in large companies compared to the ones imposed by organizations. The need to conform to them influences employees’ values, as adhering to organizational norms and values can lead to a sense of security and harmonious cooperation within the team. Sometimes employees become conformists, treating the organization as the most important goal and subordinating their values to it. Secondly, we assume that in larger companies, ethical climate perception may be determined by more self-centered and egoistic values and the welfare of individuals, rather than by altruistic and prosocial values, focused on collective well-being. However, these hypotheses require further research.

The Polish language version of the ECQ has satisfactory psychometric properties, even better than the original one. The results of confirmatory factor analysis (CFA) made it possible to demonstrate good internal validity of the modified version of the ECQ. Like in the original version, the five-factor solution was supported as having the best fit to the data. The heterotrait-monotrait ratio of correlations (HTMT) method supported good discriminant validity of the factors being part of the ECQ, showing that the ratio for all pairs of types of ethical organizational climate did not exceed 0.9^23^. Also, following Bagozzi & Heatherton’s^25^ criteria of convergent validity, the magnitude of most factor loadings was more than 0.50, the composite reliability value was more than 0.70, and the average variance extracted exceeded 0.50. One exception was the instrumental climate, but the value obtained was slightly different from the required standard.

All subscales, in comparison to the original version, had a higher Cronbach’s alpha coefficient confirming the good reliability of this measure, and it was additionally supported by the composite reliability procedure. In the case of two factors, namely the caring ethical climate and the instrumental ethical climate, it could be a consequence of the limitation of items from seven to four on each of them, but this did not concern the independence ethical climate, where the original item structure was retained. Despite this, in the Polish conditions, the independence ethical climate dimension has good reliability compared to its original scale counterpart. The original item structure of the ECQ was modified because three items, indicators of both the caring climate and the instrumental climate, did not load these factors at least on the reference admissible level. What is worth emphasizing is that the value of the correlation coefficient between the law and code climate and the rules climate was strong compared to the moderate one in the original version of the ECQ. Also, in a sample of Taiwanese nurses, this relationship was only weak^35,36^, moderate among employees from Hong Kong^10^, and not significant in Danish companies^38^, which implies the sensitivity of this measure to cultural influences^33^.

The above suggests that in Polish realities, policies, practices, and procedures, which have ethical connotations and are the basis of decision-making, the rules climate is strongly influenced by a cosmopolitan point of view, taking into account the ethical values derived from external and universal ethical standards, such as the Bible or the law^5,8^. It seems reasonable because Poles are a very religiously involved nation^39^ and religion has an impact on many areas of life. We believe that in Poland, religious socialization, which is focused on altruistic values, can be a possible factor responsible for a more than twice stronger positive correlation between the independence and caring climates compared to the original version^4^, whereas among Danish employees, it was non-significant^39^, just like in the case of Hong Kong companies^10^ and managers from Russia^40^. This means that the independence climate is based on individual values and beliefs, and moral code among Polish employees is often focused on altruistic, non-self-centered attitudes as attributes characteristic of a caring climate. These altruistic values ​​contradict the proposal of the instrumental climate, which, in opposition to them, emphasizes egoistic and self-centered motives and concentrates on driving individual, non-collective well-being. The altruistic approach as a probable result of religious socialization among Polish employees avoiding egoistic desires and treating them as undesirable is also seen in the negative correlation between individuals’ perception of the caring and instrumental climates, which was not found in the study by Victor and Cullen^4^ where those variables were unrelated.

Some limitations of this study should be addressed. The research was not preceded by a pilot study. The generalizability of the outcomes is limited because the research sample was not randomly selected. In both research samples, the participants were relatively young. Additionally, in second sample, the vast majority were women. A single-item measure was used to verify job satisfaction. Besides the advantages, this solution has some methodological limitations, especially regarding potential problems with reliability^41^. Additionally, one general question about job satisfaction does not encompass all aspects of satisfaction with work, such as satisfaction with supervisors, workmates, atmosphere, promotion, or salary. Finally, the lack of division of the study samples into two groups, which would allow for better coverage of discriminant validity, implies the need to conduct research in a longitudinal design in the future.

In conclusion, keeping in mind the limitations of this study mentioned above, and that the Polish version of the ECQ may not apply to all Polish organizations, we believe that the presented version of the questionnaire is a valid and reliable measure and that it can be a useful tool for verifying manifestations of the ethical climate as perceived by employees of several companies in Poland. Our study has shown that the results obtained in the survey that used the questionnaire can be a good predictor of employees’ well-being and attitudes towards the organization.

## Data Availability

The data presented in this study are available on request from the corresponding author.
